# Impact of Orthodontic Decompensation on Bone Insertion

**DOI:** 10.1155/2014/341752

**Published:** 2014-11-10

**Authors:** Fabio Pinto Guedes, Leopoldino Capelozza Filho, Daniela Gamba Garib, Hugo Nary Filho, Evandro José Borgo, Mauricio de Almeida Cardoso

**Affiliations:** ^1^University of Sagrado Coração (USC), Bauru, SP, Brazil; ^2^Graduation and Post-Graduation Program, University of Sagrado Coração (USC), Bauru, SP, Brazil; ^3^Bauru School of Dentistry and Hospital for Rehabilitation of Craniofacial Anomalies, University of São Paulo, Bauru, SP, Brazil; ^4^Graduation and Post-Graduation Program, University of Sagrado Coração (USC) and Branemark Institute, Bauru, SP, Brazil; ^5^São Leopoldo Mandic College, Campinas, SP, Brazil

## Abstract

There has always been concern in determining the relationship between orthodontic tooth movement and the consequent biological costs to the periodontium and tooth root. The possibility of evaluating the tooth and bone morphology by CBCT allows more accurate analysis of qualitative and quantitative aspects of these processes. This paper presents a case report of a 20-year-old male patient with Class III malocclusion and hyperdivergent facial pattern, who was surgically treated. A significant amount of labial movement of mandibular incisors was performed during orthodontic treatment before surgery. CBCT was used for evaluation of buccal and lingual bone plates before and after tooth decompensation. The changes in the bone insertion level of maxillary and mandibular incisors in the present case encourage a reflection on the treatment protocol in individuals with dentoskeletal discrepancies.

## 1. Introduction

The direct relationship between orthodontic movement and the biological cost to periodontal tissues [1] and roots [2] of involved teeth encourages a reflection on the conventionally performed therapeutic procedures. The advent of cone beam computed tomography enabled a precise characterization of root morphology [2–5], alveolar bone, and the supporting periodontal tissue of each tooth individually [2, 6–13].

Patients with dentoskeletal deformities require buccolingual movements of incisors for surgical treatment (decompensation) or comprehensive orthodontics (compensation). In these patients, greater attention is required in planning buccolingual movements of the maxillary and mandibular anterior teeth. Tooth movement beyond the limits of the alveolar bone may cause buccal dehiscences which may predispose to gingival recession in the long term. Both buccal and lingual bone plates of mandibular incisors are very thin [14]. This concern is even greater when there is both sagittal and vertical skeletal involvement, as in skeletal Class III patients with excessive vertical facial dimension where the mandibular symphysis and alveolar ridge are even thinner [1, 7, 10, 12]. From this perspective, in order to plan orthodontic decompensation of mandibular incisors, in Class III hyperdivergent patients, the orthodontist should consider, besides the amount of incisor crowding, the gingival biotype and the effects of labial incisor movement on the buccal and lingual bone plates.

This report evaluated the effects of orthodontic decompensation on the lingual and buccal bone plates, evaluated by CBCT, in a young adult patient with Class III malocclusion and hyperdivergent pattern, who was surgically treated. A critical analysis of the cost benefit of the decompensation protocol for patients with dentoskeletal discrepancies was performed.

## 2. Case Report

**Figure 1:**
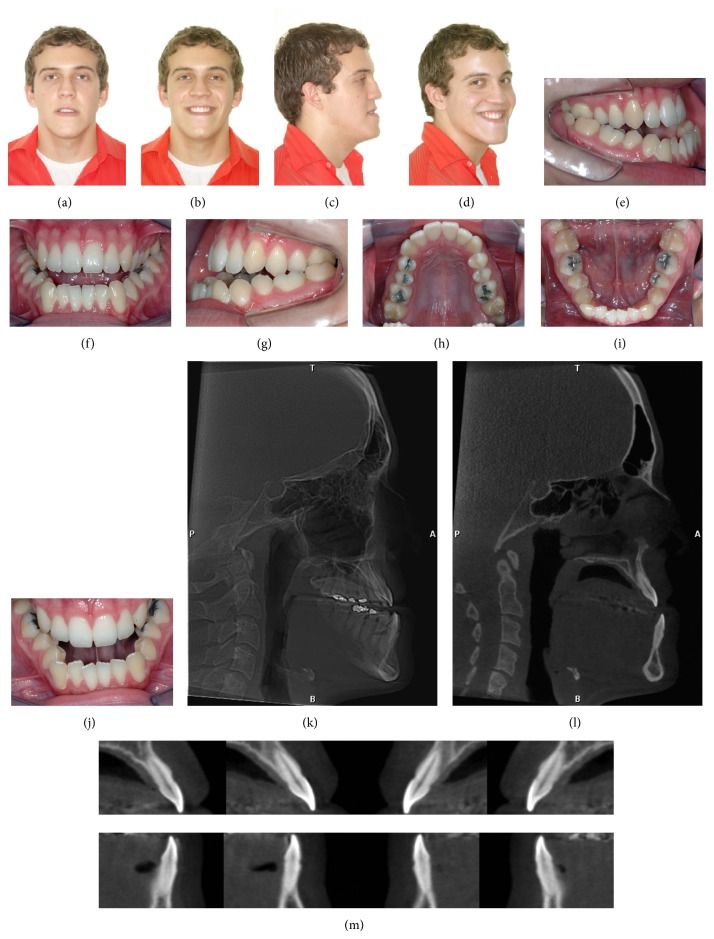
Pretreatment facial (a)–(d) and intraoral photographs (e)–(j) and CBTC (k)–(m).

A male patient aged 20.5 years sought orthodontic treatment with the chief complaint of facial disharmony. Facial analysis revealed a Class III skeletal pattern with severe mandibular prognathism and vertical excess (Figures 1(a)–1(d)). Analysis of occlusion evidenced Class III interarch relationship and anterior and posterior crossbite with a negative overjet of 6 mm. Severe tooth compensation of mandibular teeth (lingual inclination of anterior and posterior teeth) and severe mandibular anterior crowding were observed (Figures 1(e)–1(j)). The analysis of initial CBCT images (Figures 1(k)–1(m)) showed very thin buccal and lingual bone plates in the maxillary and especially mandibular incisors. A CBCT exam was acquired before orthodontic treatment, replacing the conventional extraoral radiographs.

The combined orthodontic and surgical treatment was planned. The prognosis was regular considering the magnitude of the skeletal discrepancy and the amount of required buccal movement of mandibular incisors in a thin mandibular symphysis.

The therapeutic goals were to give the patient a more balanced face and better esthetic and functional occlusion. For that purpose, the decompensation orthodontics intended to increase the negative overjet to an extent enough to allow sagittal skeletal correction. In other words, before surgery, the orthodontic comprehensive treatment aimed at aligning and leveling teeth, avoiding protrusion in the maxillary arch and promoting protrusion in the mandibular arch.

The initial dental cast manipulation showed that the posterior crossbite was mainly a consequence of a Class III anteroposterior interarch relationship. Occluding the dental casts in Class I showed that maxillary constriction was mild and a small amount of dentoalveolar expansion was not necessary to achieve an adequate transversal interarch relationship.

Comprehensive orthodontic treatment was conducted using preadjusted brackets. Dentoalveolar expansion was performed with expanded maxillary archwires supported on the second molars, which in turn would be anchored by a welded transpalatal bar fabricated with 1.2 mm round wire.

**Figure 2:**
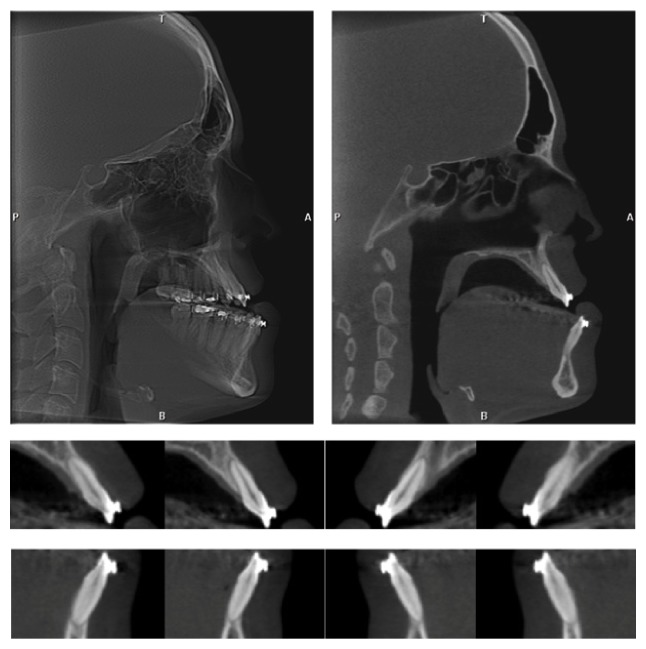
Postdecompensation CBTC.

**Figure 3:**
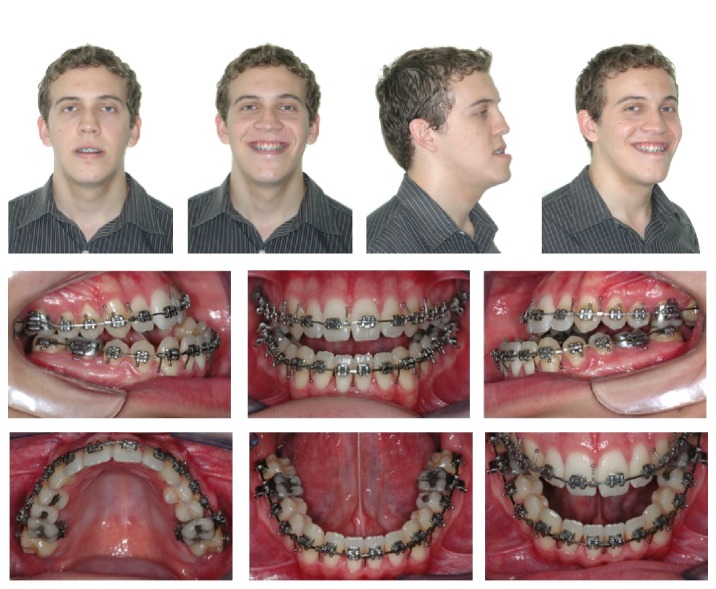
Postdecompensation facial and intraoral photographs.

After alignment and leveling and 30 days after placement of 0.019′′ × 0.025′′ archwires, the patient underwent a second CBCT scan in the same machine to evaluate the biological effects of orthodontic decompensation (Figure 2). The same exam was used for planning the orthognathic surgery. At this stage, the facial profile and occlusal relationships were worse than in the initial stage (Figure 3).

The surgical treatment planning included maxillary advancement and impaction, mandibular setback and counterclockwise rotation, and mentoplasty to reduce the anterior facial height.

**Figure 4:**
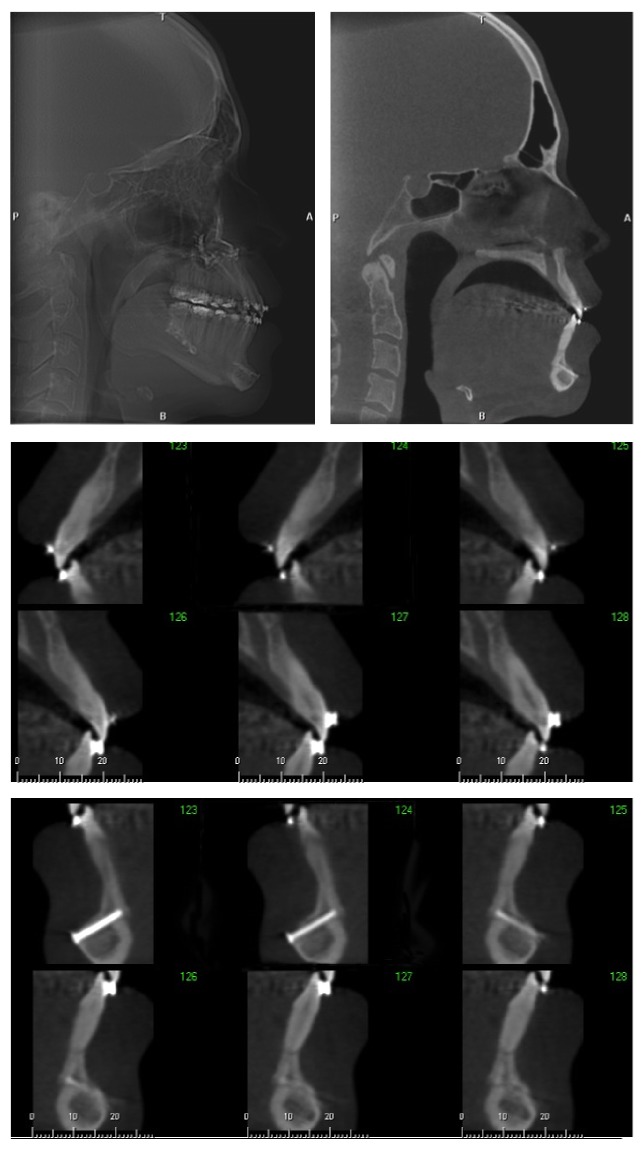
Final CBCT.

**Figure 5:**
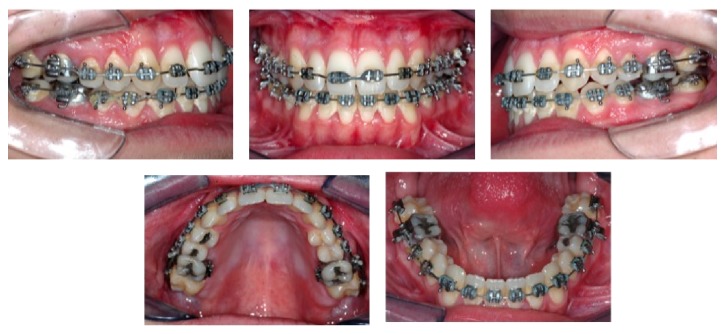
Intraoral photographs, last phase.

**Figure 6:**
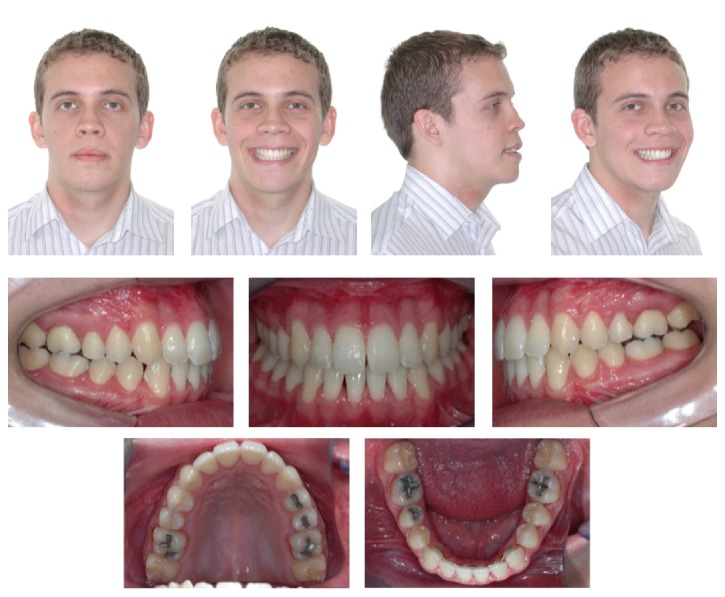
Final facial and intraoral photographs.

**Figure 7:**
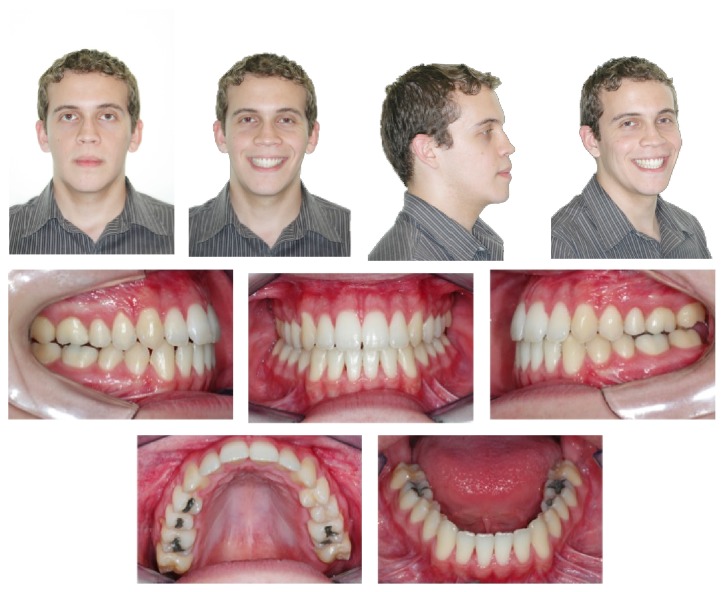
30-month posttreatment facial and intraoral photographs.

Four months after surgery, the final CBCT was requested to evaluate the condyle morphology and evaluate postsurgical dentoskeletal changes (Figure 4). The postsurgical orthodontics involved finalization bends in the archwires and utilization of Class III intermaxillary elastics (Figure 5). After six months of surgery stabilization, the fixed appliance was removed and Hawley plate and 3 × 3 mandibular retainers were placed (Figure 6). The facial and occlusal results remained stable 30 months after removal of appliances (Figure 7), including the clinical periodontal conditions.

All CBCT exams were obtained on the machine i-CAT (Imaging Sciences International, Hatfield, USA) set at the following parameters: 120KvP, 8mA, exposure time of 40 seconds, “extended face” protocol with 22 cm of FOV, and voxel of 0.4 mm. In the following, the images in DICOM—original images obtained on tomographies—were transferred to a conventional computer for manipulation in the software InVivoDental 5. Initial and postsurgical CBCT images were used for measuring the level of buccal and lingual bone plates, following the method proposed by Kim et al. [7]. The root length of maxillary and mandibular incisors was measured in the same exams.

**Figure 8:**
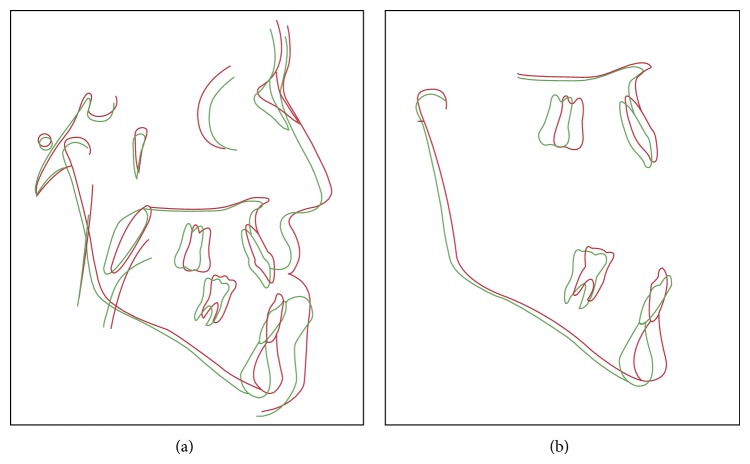
Cephalometric superimposition*: red line*: pretreatment;* green line*: postdecompensation.

**Figure 9:**
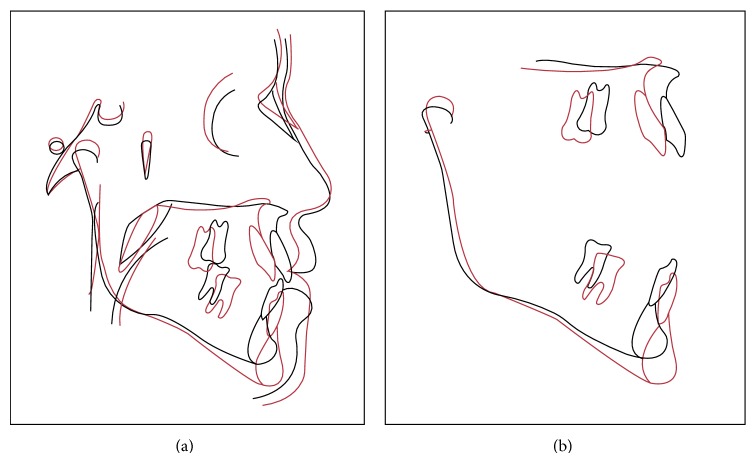
Cephalometric superimposition:* red line*: pretreatment;* black line*: posttreatment.

Cephalometric features of the case are described in Table 1, at pretreatment phase (T1), after tooth decompensation (T2), and posttreatment (T3) (Figures 8 and 9).

The comparison between initial and postsurgical CBCT images revealed the development of buccal and lingual dehiscences in the incisors in both dental arches. The mean apical migration of the buccal alveolar crest in the incisor region was 1.93 mm (range from 0.89 to 3.88) in the maxillary arch and 1.16 mm (range from 0.47 to 2.91) in the mandibular arch (Table 2). The mean apical migration of the lingual alveolar crest of the incisors was 1.76 mm (range from 0.58 to 3.87) in the maxillary arch and 1.96 mm (range from 1.07 to 3.5) in the mandibular arch (Table 3). Mild apical root resorption was observed after surgery. The root length of incisors exhibited a mean reduction of 0.25 mm (range from 0.15 to 0.45) in the maxillary arch and 1.02 mm (range from 0.46 to 1.37) in the mandibular arch (Table 4).

## 3. Discussion

The surgical orthodontic treatment is indicated when the patient present a significant facial or dentoalveolar deformities, in which the orthodontic and/or orthopedic treatment alone would not achieve satisfactory results [15–17]. For preparing Class III surgical cases for orthognathic surgery, orthodontic decompensation of the incisors is necessary [1, 18]. The main purpose of orthodontic decompensation in Class III cases is creating a negative overjet, permitting the surgical correction of sagittal discrepancies. In general, maxillary incisors are tipped lingually while the mandibular incisors are tipped buccally. Maxillary premolar extractions may be necessary for accomplishing these goals. The treatment planning should be performed in collaboration with the maxillofacial surgeon to define the magnitude of decompensation necessary for each case [16, 19]. In the present case, it was decided not to extract the maxillary premolars because the maxillary dental arch did not present significant tooth-size discrepancies and notwithstanding presented a dentoalveolar constriction. In the mandibular arch, despite the severe tooth-size discrepancy, the mandibular incisors presented a significant lingual inclination.

In the present case, changes were observed in the bone attachment level both in the buccal and lingual aspects of maxillary and mandibular incisors, after presurgical orthodontic treatment (Tables 2 and 3). It should be highlighted that the greatest bone dehiscences were observed on the lingual aspect of mandibular incisors. This corroborates the results of Kim et al. [7], who observed a bone loss of 2.8 mm and 3.8 mm in the maxillary central incisors, respectively, for the buccal and lingual aspects. In the mandibular arch, the central incisors presented a mean bone loss of 6.8 mm and 8.1 mm for the buccal and lingual aspects, respectively [7]. Sarikaya et al. [20] conducted a study to evaluate the alveolar bone repercussion of 19 patients with biprotrusion treated with extraction of four premolars and retraction of anterior teeth by means of CT. The results revealed important changes in the alveolar bone thickness and level, especially in the mandibular arch. The authors concluded that the risk of adverse effects as bone dehiscence may be present during retraction of maxillary and mandibular anterior teeth [20]. These periodontal side effects had been previously observed in some animal studies that demonstrated that tooth movement in buccal direction may cause an increase in the distance between the cementoenamel junction and the bone crest [21, 22].

It is important to highlight the ability of CBCT to provide a better observation of dental, skeletal, and especially periodontal structures, compared to conventional radiographs. This allows a more accurate diagnosis and consequently the prognosis, therapeutic goal, and treatment planning coincident with the individual characteristics of each patient. Therefore, understanding the patient and his or her limitations is necessary for a better understanding of the cost-benefit relationship of the treatment proposed [6, 7, 12, 14]. The literature reveals that despite the limitations of conventional radiographs, especially the lateral cephalogram, some authors investigated the relationship between orthodontic movement and the inherent biological costs. The evidence presented by Handelman in 1996 [1] seems to be confirmed on tomographic images.

Studies have been conducted to evaluate the precision and accuracy of quantitative analysis of dental and periodontal structures in CBCT [3, 8, 10, 11, 23]. Concerning the quality of images obtained by cone beam computed tomography, in the present case, the voxel—directly related specification—of 0.4 mm allowed good observation of the bone morphology and consequently the measurement of* BABL*,* LABL,* and* RL*. It should be mentioned that some factors—quality of image and thickness of bone plates—may influence the accuracy of measurements and consequently the interpretation of results. Therefore, protocols for achievement of images and measurements should be established [23].

The number of specific software programs developed to optimize the manipulation of images in DICOM—*Digital Imaging and Communications in Medicine*—obtained on tomographies is increasing. The method described by Kim et al. [7] and presently adapted for utilization on the software* InVivoDental 5* (Anatomage, San Jose, CA) considered all pertinent variables to evaluate the bone insertion level and root length. Utilization of the cementoenamel junction (CEJ) as reference provided a stable landmark. This way, the loss of root length inherent to orthodontic treatment did not interfere with evaluation of alveolar bone level after orthodontic treatment.

The buccolingual decompensation movement, especially of mandibular incisors, can surpass the biological limits and lead to resorption of bone plates [12, 14]. The present results suggest that the extent of loss of alveolar bone insertion and root length may be related to the magnitude of crowding and orthodontic decompensation. It should be mentioned that as the teeth presented significant rotations preoperatively, with buccal and lingual aspects turned toward the interproximal bone crests, the measurements of bone insertion levels were probably influenced.

Even though buccal and lingual bone loss was observed, the patient did not present clinically important periodontal changes. This suggests that the thickness of keratinized gingiva, presence of visible plaque, and previous gingival inflammation would be the most important predictive factors related to the risk of occurrence gingival recessions.

These findings showing loss of buccal and lingual attachment should be considered when planning buccolingual movements of the incisors during decompensation. The bone dehiscences observed in this case seem to be related to the quantity of crowding and incisor movements in the buccolingual direction. Patients with indication for orthognathic surgery followed during the growth period should have their mandibular crowding minimized by interrupting the natural compensatory movement (with a limiting agent as a lingual arch) and/or by reducing the tooth volume with extractions. In surgical adult patients, with Class III facial pattern, common sense is necessary for the team, including orthodontist and surgeon, to define the minimum decompensation to achieve the treatment objectives, including the balance, the skeletal relationships, and an adequate facial impact. Additionally, considering the gingival phenotype and the preexisting periodontal condition should be considered initially to define the limits of incisor movements.

The decompensation movement before orthognathic surgery had an influence on the buccal and lingual bone insertion levels of the incisors. Therefore, during orthodontic decompensation in Class III patients, the buccal movement should be restricted to a minimal amount that permits the accomplishment of anteroposterior jaw movements according to the surgical treatment planning.

## Figures and Tables

**Table 1:** Cephalometric summary.

Cephalometric summary			
	Predecompensation	Postdecompensation	Posttreatment
SNA (°)	72.36	73.15	82.67
SNB (°)	79.90	79.92	80.51
ANB (°)	−7.54	−6.77	2.15
SN.Gn (°)	69.52	69.84	65.78
SN.GoMe (°)	41.64	42.94	33.14
1/./1 (°)	146.29	127.13	138.58
1/.NS (°)	102.83	104.43	100.20
1/.NA (°)	30.47	31.28	17.54
1/-NA (mm)	8.27	8.46	6.07
/1.NB (°)	10.78	28.35	21.73
/1-NB (mm)	0.71	6.51	4.88
FMIA (°)	84.49	65.99	71.52
FMA (°)	26.27	28.51	20.40
IMPA (°)	69.25	85.50	88.08

**Table 2:** Pre- and postdecompensation buccal alveolar bone level values.

BABL			
(Buccal alveolar bone level, mm)			
	Predecompensation	Postdecompensation	Result
12	2,36	3,84	−1,48
11	1,75	5,63	−3,88
21	1,18	2,65	−1,47
22	2,36	3,25	−0,89
32	11,29	11,76	−0,47
31	8,87	9,66	−0,79
41	8,87	9,36	−0,49
42	7,65	10,56	−2,91

**Table 3:** Pre- and postdecompensation lingual alveolar bone level values.

LABL			
(Lingual alveolar bone level, mm)			
	Predecompensation	Postdecompensation	Result
12	2,06	5,93	−3,87
11	2,05	2,63	−0,58
21	1,77	2,93	−1,16
22	2,07	3,53	−1,46
32	8,26	11,76	−3,5
31	10,69	11,76	−1,07
41	9,48	10,56	−1,08
42	7,35	10,56	−3,21

**Table 4:** Pre- and postdecompensation root length values.

RL			
(Root length, mm)			
	Predecompensation	Postdecompensation	Result
12	13,59	13,44	−0,15
11	14,89	14,64	−0,25
21	14,19	14,04	−0,15
22	14,19	13,74	−0,45
32	14,93	13,86	−1,07
31	13,73	12,36	−1,37
41	13,12	12,66	−0,46
42	14,63	13,44	−1,19
